# Spiking CMOS-NVM mixed-signal neuromorphic ConvNet with circuit- and training-optimized temporal subsampling

**DOI:** 10.3389/fnins.2023.1177592

**Published:** 2023-07-18

**Authors:** Anuar Dorzhigulov, Vishal Saxena

**Affiliations:** AMPIC Lab, Department of Electrical and Electronic Engineering, University of Delaware, Newark, DE, United States

**Keywords:** spiking neural networks, convolutional neural networks, MaxPool, neuromorphic circuits, mixed-signal circuits

## Abstract

We increasingly rely on deep learning algorithms to process colossal amount of unstructured visual data. Commonly, these deep learning algorithms are deployed as software models on digital hardware, predominantly in data centers. Intrinsic high energy consumption of Cloud-based deployment of deep neural networks (DNNs) inspired researchers to look for alternatives, resulting in a high interest in Spiking Neural Networks (SNNs) and dedicated mixed-signal neuromorphic hardware. As a result, there is an emerging challenge to transfer DNN architecture functionality to energy-efficient spiking non-volatile memory (NVM)-based hardware with minimal loss in the accuracy of visual data processing. Convolutional Neural Network (CNN) is the staple choice of DNN for visual data processing. However, the lack of analog-friendly spiking implementations and alternatives for some core CNN functions, such as MaxPool, hinders the conversion of CNNs into the spike domain, thus hampering neuromorphic hardware development. To address this gap, in this work, we propose MaxPool with temporal multiplexing for Spiking CNNs (SCNNs), which is amenable for implementation in mixed-signal circuits. In this work, we leverage the temporal dynamics of internal membrane potential of Integrate & Fire neurons to enable MaxPool decision-making in the spiking domain. The proposed MaxPool models are implemented and tested within the SCNN architecture using a modified version of the aihwkit framework, a PyTorch-based toolkit for modeling and simulating hardware-based neural networks. The proposed spiking MaxPool scheme can decide even before the complete spatiotemporal input is applied, thus selectively trading off latency with accuracy. It is observed that by allocating just 10% of the spatiotemporal input window for a pooling decision, the proposed spiking MaxPool achieves up to 61.74% accuracy with a 2-bit weight resolution in the CIFAR10 dataset classification task after training with back propagation, with only about 1% performance drop compared to 62.78% accuracy of the 100% spatiotemporal window case with the 2-bit weight resolution to reflect foundry-integrated ReRAM limitations. In addition, we propose the realization of one of the proposed spiking MaxPool techniques in an NVM crossbar array along with periphery circuits designed in a 130nm CMOS technology. The energy-efficiency estimation results show competitive performance compared to recent neuromorphic chip designs.

## 1. Introduction

Our contemporary society generates an enormous amount of sensor data, often unstructured, with the pervasive use of smartphones, tablets, cameras, and emerging smart vehicles. As a result, this vast amount of digital data requires transfer, storage, and processing to make sense of it. Deep Neural Networks (DNNs) have found unprecedented success in inferences based on unstructured data. Consequently, Convolutional Neural Networks (CNNs) have become the staple architecture for visual data processing tasks, such as object detection and image classification. DNNs, such as CNNs, are commonly implemented in software and deployed on graphic processing units (GPUs) or neural network accelerator application-specific integrated circuits (ASICs). However, these approaches, although powerful, run into memory access bottlenecks, where the energy required to access and transfer NN data is several magnitudes higher than the energy needed for actual computing. The operation of Vector-Matrix Multiplication (VMM) is essential for DNNs. However, in a commonly utilized von Neumann architecture, VMM could consume up to × 200 more energy to move data between the memory and processing units compared to the energy required for the data processing itself (Sze, 2020).

Merging memory and compute units is a potential solution to mitigate this energy bottleneck of von Neumann architectures. Such an architectural design approach is called In-Memory Computing or Compute-in-Memory (CiM) (Ielmini and Wong, 2018; Verma et al., 2019; Sebastian et al., 2020). The emergence of non-volatile memory (NVM) devices and their foundry integration has allowed circuit designers to consider implementating core DNN functions as energy-optimized CiM circuits. While in-memory computing prototypes utilize volatile DRAM and SRAM memories (Su et al., 2021; Xie et al., 2021), realizing it in NVM arrays is desirable to implement persistent weights and higher weight density. Emerging NVM devices promise low switching energy, higher density, and endurance compared to traditional devices, such as FLASH. Resistive RAM (ReRAM) or memristors, phase change RAM (PCRAM), and ferroelectric FET (FeFET) are the most notorious examples of emerging NVM devices, recently getting traction in the CiM research community.

In such mixed-signal architectures, NVM cells are arranged into crossbar or cross-point arrays. The conductance of the NVM cell serves as the analog weight, and applying a voltage across the NVM cell results in output currents, which, if summed, act as the VMM result in the analog domain (Saxena, 2021a). These NVM devices are preferred in a crossbar array with a select transistor, i.e., using the 1T1R or 2T2R cells. In fact, select transistors are essential for sneak-path current mitigation during electroforming and program/erase operations by limiting current in the memory device (Li T. et al., 2017). Consequently, the current vs. voltage (I–V) characteristics of the entire memory cell depend on the transistor's I–V curves. The resulting compound 1T1R cell exhibits nonlinear I-V characteristics, as discussed later in Section 2.1. Furthermore, this nonlinearity depends on process, voltage, and temperature (PVT). The weighting operation is essentially an analog multiplication, and the PVT-variable nonlinearity produces an imprecise multiplication, which eventually degrades the classification accuracy of the hardware DNN (Guo et al., 2017). Alternatively, encoding the analog input as a bi-level sequence of pulses or spikes alleviates the effect of such nonlinearity. As a result, spike-based or Spiking Neural Networks (SNNs) are attractive for realizing neuromorphic computing hardware architectures which leverage mixed-signal in-memory computing. As elucidated later, SNNs allow precise neural network computations with low-precision weights or synapses, in addition to low-power event-driven circuit realization (Saxena, 2021a). These advantages of SNNs over other mixed-signal neuromorphic architectures merit investigating into the spike-based realization of traditional deep architectures, such as CNN, in NVM crossbar arrays.

**Figure 1:**
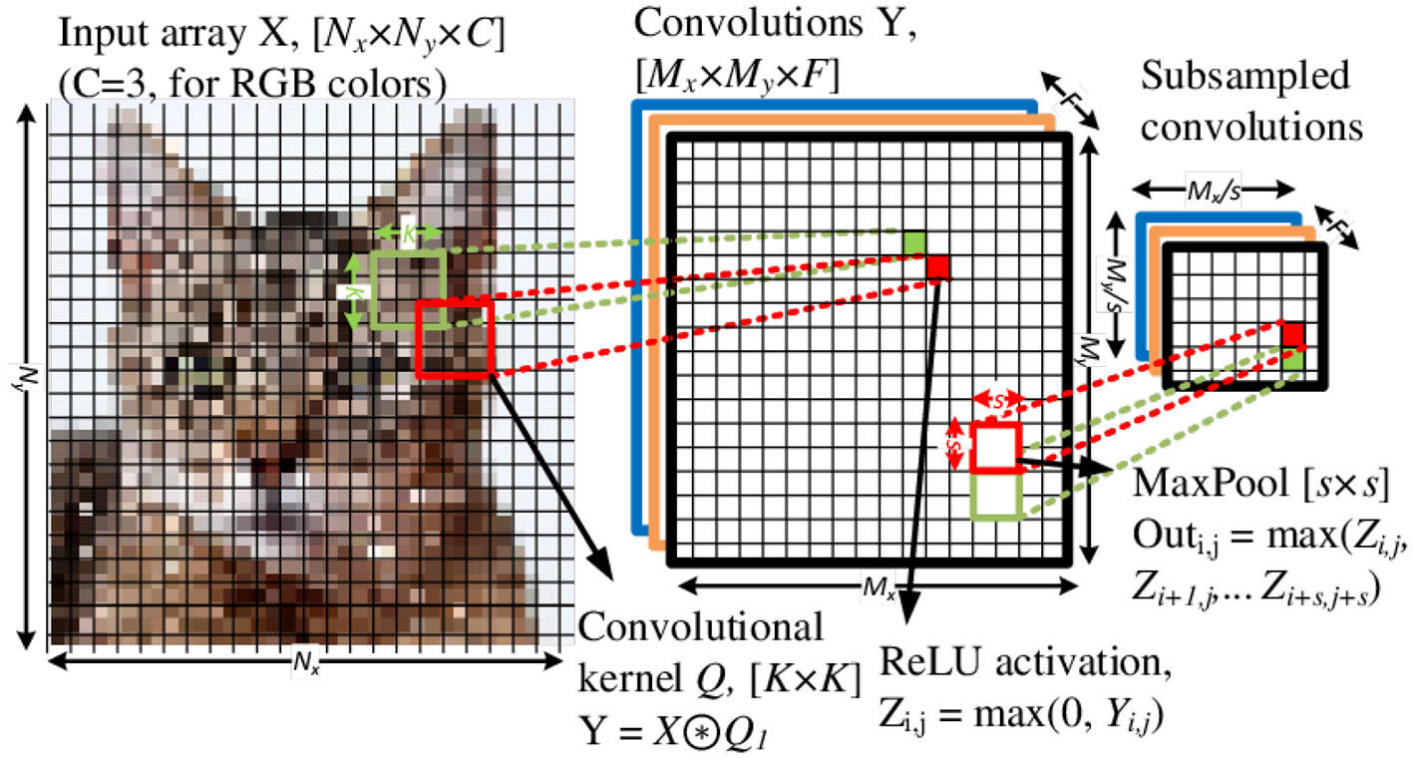
A two-dimensional (2D) convolutional layer comprised of Conv2D, ReLU, and MaxPool operations. The Conv2d and ReLU layers process an input tensor of size *N*_*x*_×*N*_*y*_×*C* using a *K*×*K* kernel and produce an output tensor of size *M*_*x*_×*M*_*y*_×*F*, where the *C* and *F* are numbers of input channels and output features, respectively. Also, here *M*_*x*/*y*_ = *N*_*x*/*y*_−*K*+1 for a stride of one and no zero padding. The Maxpool operation spatially subsamples the output tensor by a *s*×*s*.

CNN models comprise three primary mathematical/logical operations: convolution, nonlinear activation (such as rectified linear unit or ReLU), and maximum pooling (MaxPool or MP), as illustrated in Figure 1. A direct translation of CNN to in-memory computing arrays is not straightforward. Significant challenges arise from the observations: (i) using a *K*^2^×1 array to implement the *K*×*K* kernel under-utilizes the crossbar array, (ii) the translating window of the convolutional kernel is neither biologically inspired nor amenable for in-memory computing, and (iii) the CNN has to process the spike-coded inputs which are in fact spatiotemporal signals (i.e., have an additional temporal, or time, dimension). The last point makes realizing the MaxPool operation especially challenging with mixed-signal SNN hardware. Previous work experimented with alternative subsampling techniques, such as AveragePool (Lin et al., 2014; Iandola et al., 2016) and Non-overlapping Convolutional Kernel Windows (Springenberg et al., 2015), but MaxPool tends to show superior accuracy (Gopalakrishnan et al., 2020). The fundamental contribution of this work includes investigating spike-based CNN (SCNN) architecture suitable for analog mixed-signal implementation. This entails developing spike-based MaxPool operation compatible with crossbar ReRAM arrays and analog CMOS peripheral circuits. In this work, we propose and compare novel membrane potential-based pooling techniques that exploit temporal multiplexing for a significant reduction in hardware. Moreover, the proposed scheme supports native *autograd* based backpropagation training of SCNNs in PyTorch without any spike-specific methods such as surrogate gradients. The rest of this article is arranged as follows: Section 2 provides a brief introduction to CNNs using ReRAM arrays; Section 3 covers an overview of spiking neural networks and spike encoding and why they are relevant for NVM-based CNNs; Section 4 reviews prior used subsampling techniques and proposes spike-based MaxPooling algorithms for SCNNs. Section 5 presents a software pipeline to evaluate the proposed MaxPool algorithms by training the SCNN using a PyTorch-based device- and circuit-aware framework. Section 6 demonstrates a CMOS transistor-level circuit implementation of the integrate and fire (I&F) neuron with the proposed MaxPool algorithm, temporal multiplexing and circuit reuse in the NVM array. Finally, Section 7 presents a summary discussion.

## 2. Convolutional neural networks using ReRAM array

LeCun pioneered the now well-established sequential arrangement of Convolution, Nonlinear Activation Function, Pooling, Fully-connected (or dense) layers for digits recognition (LeCun et al., 1989). The superiority of CNN architectures was solidified by AlexNet winning the ImageNet 2012 challenge for large-scale visual recognition challenge (Krizhevsky et al., 2012). CNNs are excellent function approximators due to their superior image feature extraction capability. They thus have become architectures of choice for a wide range of applications, such as image recognition, video analysis, facial recognition, medical analytics, and object detection for autonomous driving (Bhatt et al., 2021).

Compared to the fully connected layers, where each neuronal connection is treated individually, CNN layers maintain spatial dependency between inputs (or pixels) by processing them as a group of neighboring spatial inputs. Each input image group (or an image patch) is processed using the same filters (or kernels) and reusing weights and biases. This spatial filtering-based approach significantly reduces the number of training parameters compared to traditional DNNs. In addition, Pooling layers (MaxPool, AveragePool) reduce the data dimensions as it flows through a CNN. As a result, the same computational resources could be used to train larger CNNs, compared to fully-connected DNNs (Wu and Gu, 2015).

**Figure 2:**
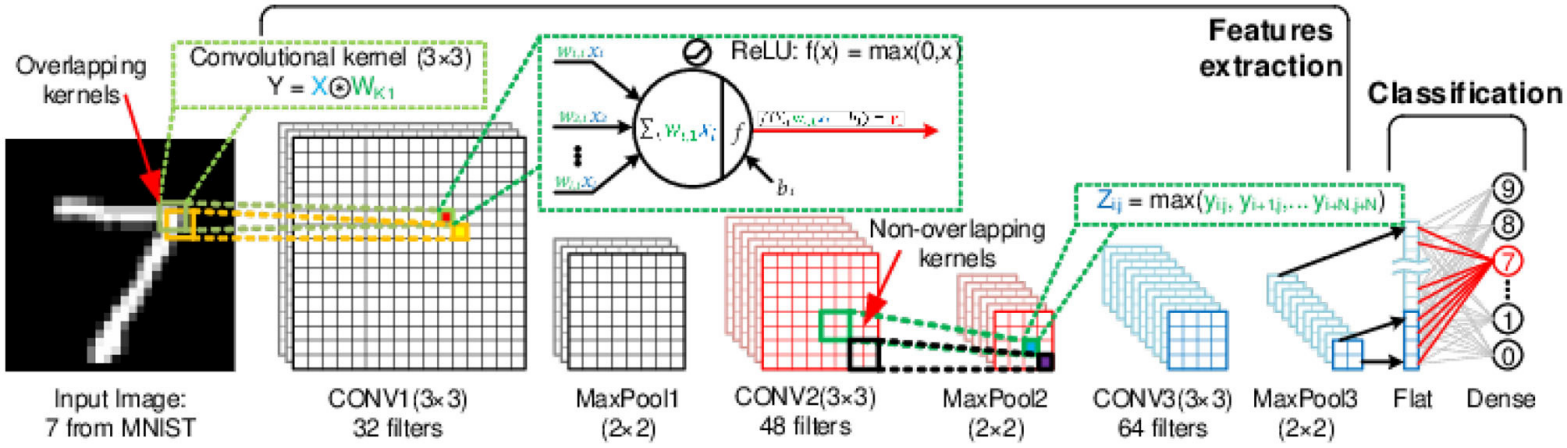
Deep CNN architecture for handwritten digit recognition from the MNIST dataset: 28 × 28 × 1-32c3-2s-48c3-2s-64c3-2s-10o. This architecture was used as a baseline for all training cases described in this manuscript. For MNIST and FashionMNIST datasets, the input size is 28 × 28 × 1 and 32 × 32 × 3 for CIFAR10.

Figure 2 illustrates the baseline CNN architecture used in this work, where hidden layers are convolutional, and the CNN's last layer(s) is fully connected (or dense). In the final output layer, each dense neuron provides confidence in its output, typically corresponding to a label in the training data. For example, in the case of MNIST (handwritten digits image classification), the output neuron corresponding to the output “7” should have the highest activation if the input stimuli is an image of the digit “7,” as shown in Figure 2. Essentially a deep CNN architecture comprises two primary sections, as depicted in Figure 1: the feature extraction section using an arrangement of convolutional stacks and the classification, or the inference, section using dense layers. Additional layers are frequently used to assist with DNN training, such as specialized initialization, batch normalization, drop-out, regularizers, and gradient boosting (Goodfellow et al., 2016; Nielsen, 2017). However, they were not considered for SCNNs in this work.

### 2.1. Mixed-signal VMM using ReRAM crossbars

Vector-Matrix Multiplications (VMMs) computations are fundamental to neural network architectures, whether fully-connected or convolutional NNs. The VMM computation is ideally expressed as


(1) $$\begin{array}{l} y_{j}=\underset{i}{\sum }w_{i,j}x_{i} \end{array}$$

where *x*_*i*_ and *y*_*j*_ are analog (or real-valued) inputs and outputs, respectively, and *w*_*i, j*_ are the neural network weights. Emerging non-volatile resistive memory arrays, such as ReRAMs, are attractive to implement VMM in the analog domain. The analog mixed-signal realization eliminates digital multipliers and adders. It promises low-power neuromorphic or Edge-AI hardware by leveraging the low program/erase energy and higher write endurance (>10^8^ cycles) of emerging NVMs. In a ReRAM crossbar array, an op-amp provides a virtual ground at the array output, so currents flowing through each branch, *I*_*j*_, follow Kirchoff's current law (KCL), as in Equation (2), resulting in a current sum at the output. Comparing Equations (1) and (2), voltage, ReRAM conductance, and current correspond to the input, weight, and weighted sum, respectively. The currents, *I*_*j*_, can be further converted to analog voltages or spikes. Moreover, weights realized using ReRAM conductance, *G*_*i, j*_, can be initialized and/or updated based on the learning algorithm employed (Wu et al., 2015b; Saxena, 2021b).


(2) $$\begin{array}{l} I_{j}=\underset{i}{\sum }G_{i,j}V_{i} \end{array}$$

**Figure 3:**
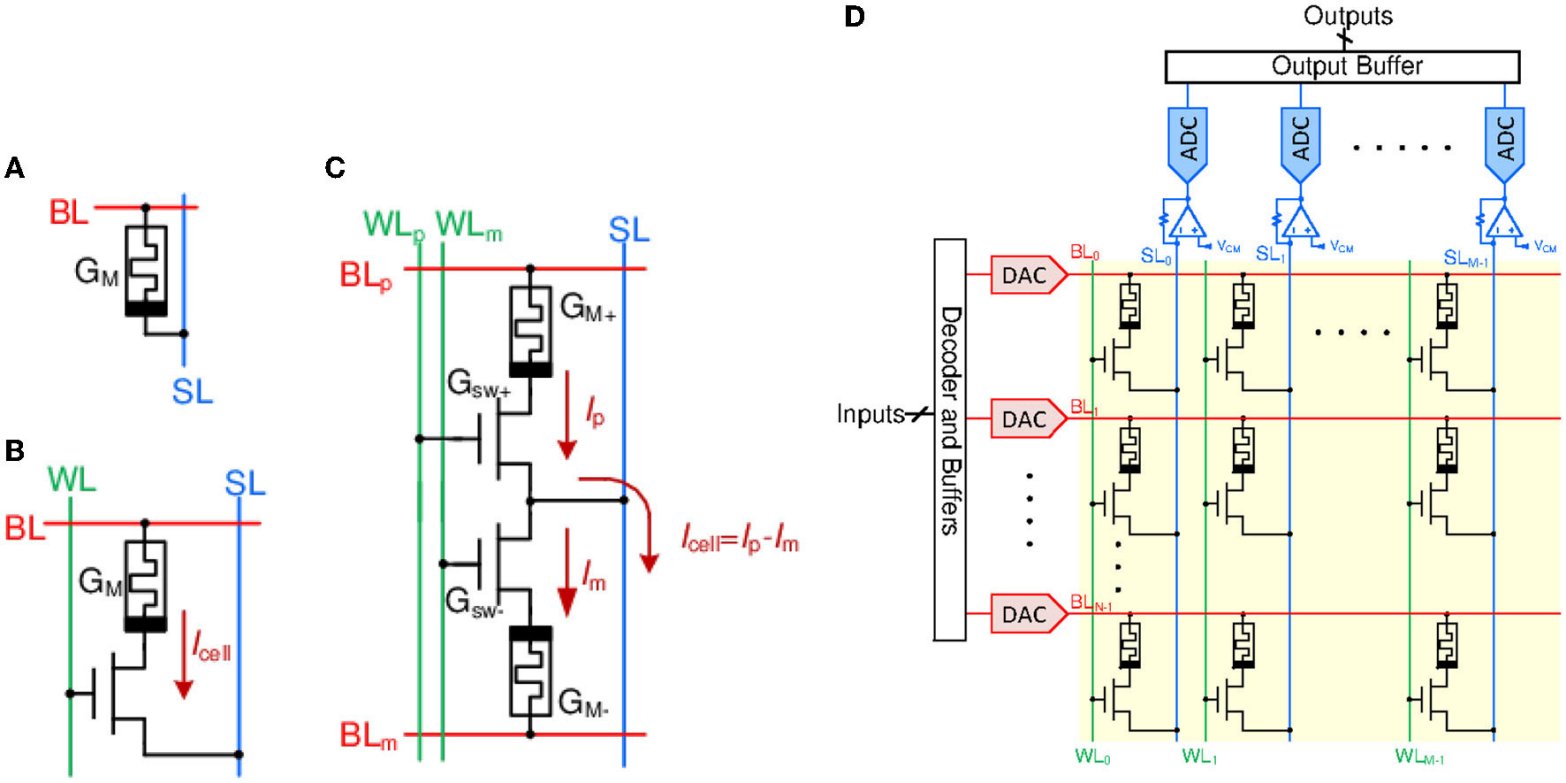
Crossbar array cell configurations: **(A)** 1R, **(B)** 1T1R, and **(C)** 2T2R. WL, SL, and BL are the WordLine, BitLine, and SelectLine, respectively. **(D)** A non-spiking VMM realized using a 1T1R crossbar array with row DACs, column transimpedance amplifiers (TIA) and ADCs. The 2T2R array for realizing signed weights will be similar but will use two BLs instead of one, one for each 1T1R cell.

Ideally, it is expected for ReRAM devices to exhibit stable multilevel cell (MLC) retention. However, practical devices exhibit limitations such as variability, low on-state resistance (high energy consumption), and state drift, detailed in Esmanhotto et al. (2020) and Saxena (2021b). Figure 3 shows canonical weight, or synapse, cell configurations. The 1T1R cell (T, select transistor; R, ReRAM device) mitigates some non-idealities and has become the preferred configuration despite its lower array density (Danial et al., 2019). Here, device isolation using the select transistor prevents sneak path currents during a read operation or inference and stabilizes the forming and the program/erase processes by limiting the current through the device.

In Figure 3B, each 1T1R cell has three terminals: BitLine (BL), shown in red, is connected to the top electrode of the cell and supplies input voltage. WordLine (WL), shown in green, is connected to the select transistor gate, enabling the required NVM cell. Source or Sense Line (SL), shown in blue, is connected to the bottom electrode and is used to read out the output current (through a virtual ground).

While a 1T1R cell realizes analog weight, a 2T2R or a similar cell is used for signed weights, as shown in Figure 3 (Liu et al., 2020). The resistance of the 1T1R cell is *R*_*cell*_ = *R*_*sw*_+*R*_*M*_, where *R*_*sw*_ and *R*_*M*_ are the transistor switch and ReRAM resistances, respectively. Here, *R*_*sw*_ depends upon the input voltage on a BL, *V*_*i*_, and the ReRAM state leading to nonlinear I-V characteristics. Consequently, the conductance and current in the 1T1R cell can be expressed as


(3) $$\begin{array}{ll} G_{cell} & =G_{sw}\left(V_{i},G_{M}\right)∥G_{M} \end{array}$$

(4) $$\begin{array}{ll} I_{cell} & =G_{sw}\left(V_{i},G_{M}\right)∥G_{M}\cdot V_{i} \end{array}$$

Here, $G_{sw}=R_{sw}^{-1}$ is the switch conductance, and $G_{M}=R_{M}^{-1}$ is the ReRAM conductance which can take binary or multilevel state values. Since the 1T1R (or 2T2R) cell realizes the analog multiplication, *w*_*i, j*_·*x*_*i*_≡*G*_*cell, ij*_·*V*_*i*_, the large-signal nonlinearity makes the weight input signal dependent. While this nonlinearity could be learned during the neural network training, the PVT-dependent variations make this untenable.

## 3. Spike encoding and spiking neural networks

Translating DNNs into a neuromorphic architecture entails mapping each neural network layer to one or more crossbar arrays. In a typical CiM VMM array (Figure 3D), the inputs to each array are applied using a WL (i.e., row) driven by a digital-to-analog converter (DAC) with a given bit resolution (e.g., eight bits). The summed analog outputs on BLs are digitized using column analog-to-digital converters (ADCs). As discussed earlier, the 1T1R (or 2T2R) cell exhibits PVT-dependent nonlinearity, which has rendered accurate mixed-signal VMMs challenging to design. Moreover, DACs and ADCs limit the energy and area efficiency of these VMMs.

Spike-based computation derives inspiration from biological brains to solve imprecise weights and energy consumption challenges. In a biological brain, inputs and activation intensities (*V*_*i*_) are mapped to temporal characteristics of individual spikes or a spike train, such as spike firing time or a spike firing rate. Spikes form a bilevel sequence that encodes the analog input, *V*_*i*_, as *s*_*i*_(*t*):


(5) $$\begin{array}{l} s_{i}\left(t\right)=\underset{n}{\sum }g\left(t-t_{i,n}\right)=g\left(t\right)⊗\underset{n}{\sum }\delta \left(t-t_{i,n}\right) \end{array}$$

where, *g*(*t*) is the spike pulse shape and *t*_*i, n*_ are the spike firing times for *n* = 0, ⋯ , *N*−1.

In a spiking neuromorphic architecture, the weights multiply by zero or one (two possible spike amplitude levels). Thus, the cell current from Equation (4) takes two possible values


(6) $$I_{cell}=\{\begin{array}{ll} 0, & \text{spike}=0\\ G_{sw}(V_{p},G_{M})∥G_{M}, & \text{spike}=1 \end{array}$$

resulting in linear weight multiplication, even with PVT variations. This alleviates the imprecision of analog multiplication of the input activation with weights. Furthermore, the input DAC and output ADC are now transformed into pre-synaptic and post-synaptic integrate-and-fire neurons, which can be realized with mixed-signal circuits for reduced power consumption (Saxena, 2021a).

### 3.1. Latency encoding

**Figure 4:**
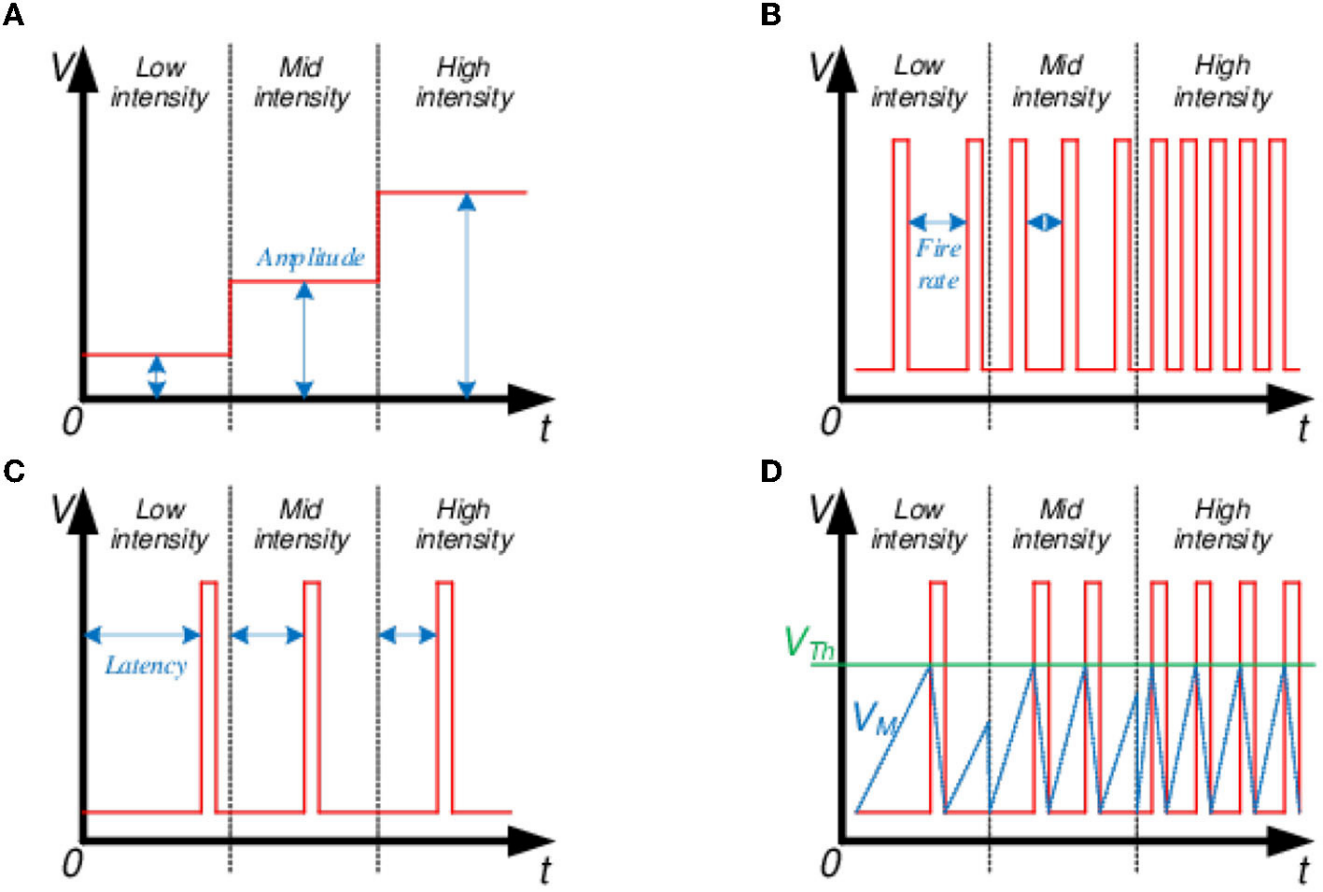
Input intensity encoding schemes: **(A)** Amplitude, **(B)** Spike latency, **(C)** Firing rate, and **(D)** Integrate and fire.

In spike latency encoding of inputs, the timing or latency of a spike corresponds to a mapped intensity (Figure 4B). Measured latency could be between two spike events (e.g., the time difference between the input and output spikes of a neuron) or at what time the spike was fired between arbitrary time-frames (e.g., sample time-frame) if fired at all. The main drawback of this encoding is the reliance on temporal information encoded in a single spike. In a hardware realization, single spike timing could be distorted by deterministic and stochastic delays or lost entirely, deteriorating data integrity (Guo et al., 2021).

### 3.2. Rate encoding

The spike firing rate represents the mapped intensity in a spike rate encoding (Figure 4C). Encoding a real-valued input as a train of spikes is a more robust scheme. Here, any instantaneous errors introduced by the hardware could be mitigated over a sufficiently long spike train due to the reliance on an average spike rate (Javanshir et al., 2022).

### 3.3. Integrate and Fire encoding

In the Integrate and Fire (I&F) encoding scheme, the input data intensity does not depend on deterministic temporal values, such as latency and rate. Instead, it is represented by the local average over a time window (Figure 4D). This local average can be obtained by integrating either the inputs over time, where the integrated value is equivalent to an I&F neuron's membrane potential (*V*_*M*_):


(7) $$\begin{array}{l} V_{M,j}\left(t\right)=\overset{t}{\underset{0}{\int }}\sum_{i=1}^{N}w_{ij}s_{i}\left(t\right)dt=\sum_{i=1}^{N}w_{ij}\overset{t}{\underset{0}{\int }}s_{i}\left(t\right)dt \end{array}$$

Once *V*_*M*_ crosses a predetermined threshold (*V*_*thr*_) at time *t*^*f*^, the neuron fires, i.e., generates an output spike and resets *V*_*M*_ to an initial resting potential (*V*_*rst*_) (Burkitt, 2006):


(8) $$\text{at }t=t_{j}^{f}\;V_{M,j}^{l}(t)\geq V_{thr,j}^{l}:\{\begin{array}{l} V_{M,j}^{l}(t)\leftarrow V_{rst}\\ s_{j}(t)\leftarrow g(t-t_{j}^{f}) \end{array}$$

For I&F encoding, we can think of *V*_*i*_ being encoded as the average of the spike train *s*_*i*_(*t*) with an encoding error ϵ:


(9) $$\begin{array}{l} V_{i}\approx \frac{1}{T}\overset{T}{\underset{0}{\int }}s_{i}\left(t\right)dt+\epsilon \end{array}$$

One common variation of the I&F neuron is a Leaky Integrate and Fire (LIF) neuron (Delorme et al., 1999). In a LIF neuron, *V*_*M*_ leaks over time, slowly pulling *V*_*M*_ to the *V*_*rst*_ level. Leakiness endows the ability to filter out low-intensity inputs and activations temporally. In addition, I&F neurons typically have a refractory period, a short time window following neuron firing reset, where *V*_*M*_ stays at *V*_*rst*_ by ignoring any input spikes. Another optional feature of the I&F neuron is lateral inhibition, which can inhibit neighboring neurons' firing if it fires itself first (Thiele et al., 2018). This lateral inhibition forms winner-take-all (WTA) neural networks commonly used for unsupervised learning (Wu et al., 2015a). This work uses the basic configuration of I&F neurons without any leakiness, refractory period, or lateral inhibition.

## 4. Sub-sampling by pooling operation

### 4.1. MaxPooling in CNNs

Pooling is a standard operation in CNNs that reduces the spatial dimensionality of data as it flows through a neural network. It is achieved by propagating a limited number of neuronal activations after a layer. Typical dimensionality reduction operations are briefly described as follows.

#### 4.1.1. MaxPooling

MaxPooling operation propagates only the strongest neuronal response to the input stimulus. In real-valued CNN architectures, where continuous or floating values represent inputs and activations, the highest amplitude of neuronal activations in a subsampling window is propagated through the MaxPool layer. Direct hardware realization of this scheme results in costly chip area and design overhead (Gopalakrishnan et al., 2020).

#### 4.1.2. Average pooling

Since mixed-signal circuit implementation of MaxPooling has significant overheads, neuromorphic circuit designers experiment with averaging or mean pooling instead. The AveragePool layer propagates forward the averaged value of all neuronal activations within a spatial pooling window (Boureau et al., 2010).

#### 4.1.3. Strided convolutional kernels

CNNs typically use unitary strides when performing convolutions. Spatial sub-sampling can be achieved using convolutional kernels with strides greater than one, which could result in non-overlapping kernels.

### 4.2. Spatial MaxPooling in SNNs

Pooling layers are indispensable for CNNs. Thus it is desirable to translate this function to the spike domain. However, MaxPooling from multiple spike trains is more complex than pooling from an array of continuous neuronal activations. As a result, some of the recent SCNN implementations favor more straightforward Pooling options. AveragePool (Wu et al., 2018; Sengupta et al., 2019; Garg et al., 2021; Yan et al., 2021) and Strided Convolutional layers (Esser et al., 2016; Patel et al., 2021) are some of the straightforward alternatives to the spiking MaxPooling. However, even in the spike domain, the MaxPooling tends to produce higher classification accuracy (Rueckauer et al., 2017) than the aforementioned alternatives. When implementing the spiking MaxPooling, researchers are drawn to several popular approaches: rate-based spike accumulation (Hu and Pfeiffer, 2016; Chen et al., 2018; Kim et al., 2020), time-to-first-spike (Masquelier and Thorpe, 2007; Zhao et al., 2014; Li J. et al., 2017; Mozafari et al., 2019), and lateral inhibition or temporal winner-take-all (Orchard et al., 2015; Lin et al., 2017).

Table 1 compares the SNN pooling techniques from prior literature. Some key observations are: (i) The membrane potential, *V*_*M*_, based MaxPooling is rarely investigated or fully utilized; (ii) The most straightforward approach is to train an ANN and then convert it into its SNN equivalent (Saxena, 2021b); (iii) Directly training an SNN with the Backprop algorithm requires additional assistance in the form of surrogate gradients (Neftci et al., 2019); (iv) MaxPooling is dynamic in the temporal domain, in other words, the winning neuron is not locked for the entire input time-frame. Since we are considering mixed-signal circuits to implement SCNNs, where we can access the integrating capacitor (Saxena, 2020) and keep the spike buffering overhead minimal (or ideally non-existent) without any additional circuit elements, such as dedicated MaxPool Neuron (Guo et al., 2020), we propose using the *V*_*M*_ potential as the MaxPooling criteria. We introduce and compare three methodologies for the spiking MaxPool.We also consider using a native PyTorch training workflow with backpropagation and *autograd* to train SCNNs with the proposed MaxPooling schemes directly. To assist with Backprop training, we are locking the MaxPool winning neuron till the end of a time-frame. In other words, the MaxPooled neuron stays the same until the input image changes. As highlighted in the Table 1, we obtained competitive results with respect to the Converted SNNs. Section 5 further elaborates on SCNN training details and results.

**Table 1:** Comparison of pooling schemes in SNNs.

**References**	**Pooling type**	**Accuracy degradation**	**Comments**
**ANN converted to SNN**			
Rueckauer et al. (2017)	MaxPool with a gating function	CIFAR10: 0.04%	MaxPool relies on firing rate estimators
Gaurav et al. (2022)	MaxPool based on input currents and MaxPool based on neuron model properties	CIFAR10: 2.5%	Based on NengoDL (Rasmussen, 2019) and dependent on Loihi hardware.
Nguyen et al. (2020)	MaxPool based on *V*_*M*_ potential and firing threshold *V*_*thr*_	CIFAR10: 5.9% with the spiking VGG-16	Digital hardware to implement the MaxPool operation by propagating fired output spikes and not selected neurons, thus making the dynamic *V*_*M*_ potential comparison unnecessary.
Guo et al. (2020)	MaxPool based on I&F neuron model	CIFAR10: 2.2% with the spiking VGG-16	Used an additional spiking neuron as a pooling controller to propagate any output spikes fired from a pooling group.
Li et al. (2022)	MaxPool based on Lateral-Inhibition pooling	CIFAR100: 0.01% with the spiking VGG-16	Used a spike calibration scheme with lateral-inhibition pooling.
Datta and Beerel (2022)	Unspecified MaxPool for SNNs	CIFAR10: 3.01%, CIFAR100: 4.59% with spiking ResNet-20	Surrogate gradient-based fine training (Rathi and Roy, 2020).
**Direct SNN training**			
Zhang J. et al. (2022)	MaxPool and AveragePool based on the spike count	MNIST: 95.4% accuracy with three Conv layers and temporal coding.	FPGA based implementation
Zhang C. et al. (2022)	MaxPool based on the temporal WTA scheme	CWRU ball bearing dataset (CWRU, 2023): 99.6% accuracy with the spiking LeNet-5 model.	SCNN trained with surrogate gradients (Neftci et al., 2019)
This work	Membrane potential (*V*_*M*_) based MaxPool techniques	CIFAR10: 2.55% for SCNN with three conv layers	Max *V*_*M*_ pooling yields the most accurate SCNN after backprop training with native *autograd*

In this work, we investigated schemes for pooling in SCNNs, which are amenable to mixed-signal circuit implementation. The specific challenge with pooling in SNNs is the latency in deciding the MaxPooled or winner neuron, i.e., the time needed to select the neuron in the spatial group whose activation will be propagated. All the neuron spikes must be buffered during the decision-making time interval to propagate the winning neuron's output spikes right after the pooling decision. Otherwise, the initial spikes of the propagated neuron are lost. On the other hand, we can access the continuous value of the membrane potential, *V*_*M*_, inside the I&F neuron to make the pooling decisions instead of using spikes. We propose and consider the following three MaxPooling schemes and present a temporal scheme to implement them in mixed-signal neuromorphic hardware efficiently.

#### 4.2.1. Maximum membrane potential

**Figure 5:**
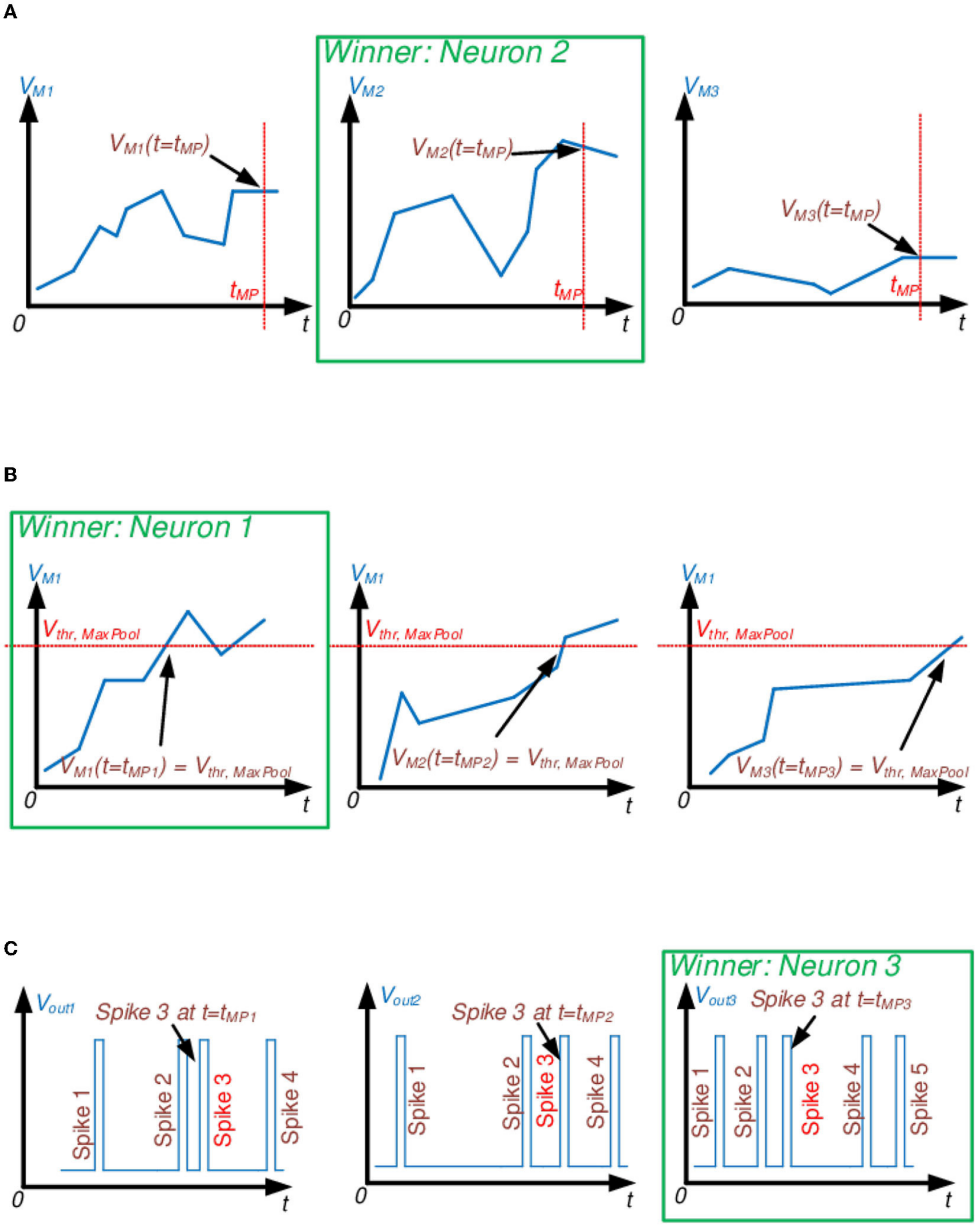
Proposed temporal MaxPool algorithms for SCNNs. Examples illustrate the 1 × 3 spatial pooling window. **(A)** Max *V*_*M*_ potential MaxPooling. Out of three given neurons, select the one with the highest membrane potential, *V*_*M*_, by the end of the MaxPool window at *t* = *t*_*MP*_. **(B)** Threshold MaxPooling. Out of three given neurons, select the one with the fastest membrane potential, *V*_*M*_, to reach *V*_*thr, MaxPool*_. **(C)** The First *N* spikes to arrive MaxPooling. Out of three given neurons, select the one with the fastest N number of spikes. In this illustration *N* = 3.

In this approach, the neuron with the highest accumulated membrane potential within a specific time range or *Pooling time window* is pooled (Figure 5A). The downside of such an approach is a requirement for additional clocking elements controlling the time period for MaxPool decisions. In addition, such a MaxPool approach will introduce a time delay for the algorithm to decide the maximum potential. The scheme can be improved if the MaxPool decision window is much shorter than the MaxPool activation window (i.e., the time period within which the selected neuron will be active or the time until the next MaxPool decision). Moreover, output spikes are not fired during the MaxPool decision time window, even if the *V*_*M*_ potential exceeds the *V*_*thr*_ threshold. However, in Section 6 of this manuscript, we propose to precharge the integrator capacitor to the winning *V*_*M*_ potential to minimize temporal data loss. If *V*_*M*_≥*V*_*thr*_, the neuron will fire an output spike once the firing circuit activates.

#### 4.2.2. MaxPool threshold

In this MaxPool algorithm, the winner neuron is determined by the timing at which its *V*_*M*_ reaches predefined *V*_*thr, MaxPool*_ (Figure 5B). The first neuron to reach it is selected for pooling. *V*_*thr, MaxPool*_ could be different from the I&F firing threshold, *V*_*thr*_, but keeping *V*_*thr, MaxPool*_ ≤ *V*_*thr*_ should keep temporal data loss minimal since no input spike contribution will be lost due to the *V*_*M*_ potential overcharging. However, if *V*_*thr*_ = *V*_*thr, MaxPool*_, this approach acts as a *Time-to-first-spike* MaxPooling (Orchard et al., 2015).

#### 4.2.3. First N spikes to arrive

This approach counts the number of spikes fired by each neuron within a group and is not necessarily based on *V*_*M*_. The first neuron to fire “N” times is pooled, and its subsequent output spikes are propagated (Figure 5C). *Time-to-first-spike* is a popular approach to implement MaxPool in SCNNs, so we were interested in exploring the idea of counting multiple spikes to improve the SCNN classification accuracy.

## 5. SCNN training using PyTorch framework

### 5.1. SCNN training

Spike-based training (or learning) algorithms are a popular topic of SNN research (Neftci et al., 2017, 2019; Saxena, 2021b). The algorithms are broadly categorized into transfer (ANN conversion), unsupervised/semi-supervised, and supervised learning. Spike Timing Dependent Plasticity (STDP) is associated with unsupervised SNN training (Vaila et al., 2019a). While these bio-inspired learning rules lend to localized learning and hardware-friendly realizations (Wu et al., 2015a; Wu and Saxena, 2018), they are limited to shallow networks with diminishing gains in terms of accuracy when multiple SCNN layers are stacked (Vaila et al., 2019b).

This work focuses on supervised learning for image classification to achieve competitive classification accuracy with deep SCNNs. In our paradigm, training is performed on GPUs with PyTorch. Since we are considering deploying SCNNs on mixed-signal neuromorphic chips, we expanded PyTorch training functionality with aihwkit to investigate the impact of NVM device non-idealities on classification accuracy. Aihwkit, or *IBM Analog Hardware Acceleration Kit*, is an open-source PyTorch-integrated toolkit for exploring the capabilities of neuromorphic devices and circuits for AI (Rasch et al., 2021). This toolkit modifies PyTorch's standard functions and training workflow to be more hardware aware. For example, the commonly used PyTorch 2D convolutional layer, *Conv2D*, is replaced by *AnalogConv2D*, where the parametric analog device model defines the behavior of the convolutional weights. The list of device-defining parameters includes the following: behavioral model (linear or exponential response to the weight update), positive and negative weight bounds, minimal and maximal weight increments, weight drift, and corrupt device probability. In addition, the forward layer-to-layer pass could be modeled with controllable ADC and DAC limitations of quantization and bounds. However, since we are investigating spiking networks, there are no digital or analog values shuttled between layers in the forward pass, only bi-level spikes, so ADC and DAC limitations are ignored in the context of SCNNs. The presented approach is well-suited for the off-chip learning paradigm since we optimize SCNN models for on-chip inference, but training is still off-chip on GPUs. On-chip learning using NVMs is a challenging problem and is elaborated upon elsewhere (Saxena, 2021b).

Image classification datasets used in this work are a collection of grayscale [MNIST (LeCun, 1998) and FasionMNIST (Xiao et al., 2017)] or colored (RGB for CIFAR10; Krizhevsky and Hinton, 2009) 2D arrays, where colors are split into channels in the dedicated third dimension. The dataset images are converted to a 4D tensor with an added temporal dimension to represent spike-domain spatiotemporal inputs. Two input conversion schemes are explored for SNN training with the proposed MaxPooling methods from Section 4.2:

***Static input scheme**:* The first scheme converts input pixel intensity into a fixed amplitude encoded signal (Figure 4A). Such an approach could be interpreted as a static input image repeated for each timestep in a time-frame or constant current levels supplied to the first hidden layer of I&F neurons to charge the membrane potential *V*_*M*_. This encoding increases spike firing frequency in otherwise low-activation neurons, thus boosting the classification accuracy of transfer-based SNNs (Rueckauer et al., 2017). We are examining if this approach improves the classification accuracy of trained SNNs.

***Spiking input scheme**:* The second scheme converts pixel intensity into a dynamic spike train, where the firing rate is proportional to the input pixel intensity (Figure 4C).

**Figure 6:**
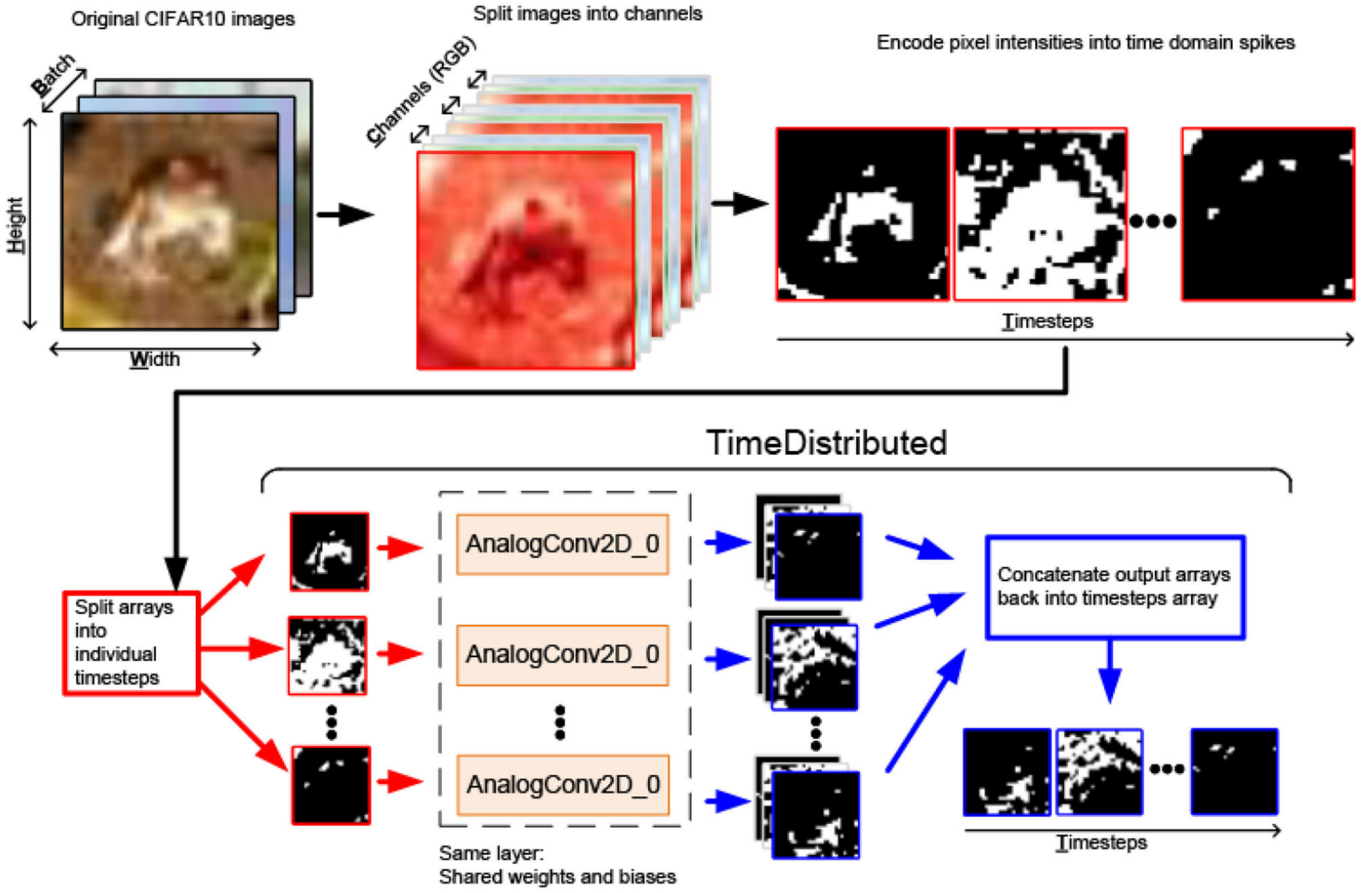
Software pipeline for SNN input conversion to BTCHW tensors and the *TimeDistributed* layer for processing them using the *AnalogConv2D* function.

Figure 6 illustrates the temporal interface implemented for 2D analog convolutional layers. PyTorch Conv2D layers, and by extension *aihwkit* AnalogConv2D layers, naturally support 4D input tensors in the BCHW format: batch, channel, height, and width. However, input images are converted into the 5D tensor BTCHW (batch, timestep, channel, height, and width).

We designed the PyTorch implementation of the *TimeDistributed* Keras wrapper (Keras, 2022) to apply the wrapped layer to every input timestep. Firstly, *TimeDistributed* splits the BTCHW input tensor into the *T* number of BCHW tensors, where *T* is the number of timesteps in a time-frame. Then each BCHW input tensor is individually fed forward through the same AnalogConv2D layer. Then the *T* number of AnalogConv2D BCHW output tensors is concatenated into the single BTCHW output tensor of the *TimeDistributed* layer. Thus, *TimeDistributed* integrates Conv2D/AnalogConv2D layers from aihwkit into the SCNN flow.

Automatic differentiation (*autograd*) is a PyTorch tool that allows convenient gradient estimation for training. Autograd estimates the gradients of a function with respect to its inputs, even if the function is defined through a complex, nested computation. This makes it easy to implement and train complex DNN models in a scalable and efficient manner (Paszke et al., 2017). However, due to the intrinsic data sparsity and spike representation, SNNs suffer from discontinuous gradients, hindering training performance, especially in latency encoding, where each neuron fires at most once per each time-frame (Neftci et al., 2019). The recent research literature proposes gradient approximation techniques such as surrogate gradient (Neftci et al., 2019; Cramer et al., 2022) to substitute gradients with non-differentiable spiking neurons to adapt Backprop for SNNs.

In our work, the weighted summation of input spikes, followed by the integrating membrane potential, *V*_*M*_, and thresholding, defines the computation graph for each I&F neuron. PyTorch autograd showed competitive results in our scheme without employing any surrogate gradient. This is unsurprising since autograd can work with a mixture of differentiable and non-differential functions, thanks to reverse-mode automatic differentiation. The reverse-mode automatic differentiation traverses the computation graph in reverse and accumulates the gradients as it goes. Thus, the gradient information is “propagated” through the computation graph, even in cases where the individual operations are not differentiable. Moreover, in our implementation, the network loss computation and weight updates are calculated only at the end of the entire time-frame, not for each timestep, thus averaging the gradient information over the entire time-frame.

SNN training and inference were performed on an Nvidia RTX 8,000 GPU with 46 GB memory on an Intel Xeon 4214R dual CPU server. They took around 22, 24, and 27 min to run one training epoch for MNIST, FashionMNIST, and CIFAR10 datasets. Each training was performed for 50 epochs. However, it has been observed that each training loss converged to the final value in < 30 training epochs for each dataset. To keep SCNN simulation time and GPU RAM load manageable, the simulations presented in this work use the time-frame length of 100 timesteps. Otherwise, doubling the number of timesteps will double the size of the computational graph, throttling the GPU. In other words, each input image from a dataset is fed forward for 100 timestep units and thus represented by 0–100 spikes. The 0–100 range roughly corresponds to the activation temporal resolution of *log*_2_(100) = 6.64 bits. The size of the time-frame can be increased for higher resolution at the cost of a longer training duration.

To estimate the effect of the proposed spiking MaxPooling techniques on classification accuracy, we compare five baseline CNN model (seen in Figure 2) implementations: *Ideal, ANN-converted-to-SNN*, and three SCNNs with the proposed spiking MaxPooling schemes trained using autograd. The *Ideal* CNN was implemented with conventional non-spiking PyTorch layers and trained with the AdaDelta optimizer. The ANN-converted-to-SNN was implemented by converting the *Ideal* CNN model into the SCNN using the *SNN toolbox* (Rueckauer et al., 2017). This toolbox was chosen for the comparison due to the near-lossless (in terms of classification accuracy) ANN-to-SNN conversion, where the dynamic gating function estimates pre-synaptic neuron firing rates to propagate spikes only from the most active neurons, thus acting as the spiking MaxPool.

Since we want to investigate if SCNN training with non-spiking inputs results in higher classification accuracy than the training with spike-coded inputs, we evaluated both for SCNNs training with the proposed MaxPooling algorithms. Table 2 shows test classification accuracy for the aforementioned cases after training with autograd.

**Table 2:** Baseline CNN model implementations testing accuracy on image classification datasets.

**CNN implementation**	**MNIST (%)**	**Fashion MNIST (%)**	**CIFAR10 (%)**
**Ideal ANN**	**99.96**	**92.66**	**82.04**
ANN-converted-to-SNN	99.70	92.61	80.56
**Autograd trained with static input**			
Max *V*_*M*_ potential	98.50	91.24	79.49
MaxPool *V*_*M*_ threshold	99.16	90.76	75.26
First three spikes to arrive	97.48	86.65	67.30
**Autograd trained with spiking input**			
Max *V*_*M*_ potential	98.26	88.16	74.19
MaxPool *V*_*M*_ threshold	98.10	87.94	71.73
First three spikes to arrive	92.38	83.98	62.38

As can be observed from these results, converting input intensities into static (i.e., non-spiking) amplitudes indeed results in higher image classification accuracy after training compared to the spike input encoding. This difference is most evident from CIFAR10 results, where the static input encoding helped to train the Max *V*_*M*_ MaxPooling SCNN with 79.49% classification accuracy, which is more than a 5% difference between the same network model trained with spiking inputs.

The second important observation is that the proposed Max *V*_*M*_-based MaxPool SCNN implementation achieves the highest classification accuracy after training out of the three proposed MaxPooling schemes. Furthermore, these results compare well with the converted model with < 1.5% degradation in classification accuracy across the three image classification tasks. Compared to the ideal real-valued ANNs results, the degradation exceeds 1.5% only for CIFAR10, where it achieves a 2.55% difference.

### 5.2. *V*_*M*_-based MaxPool with partial time window

As discussed earlier, the first scheme with Max *V*_*M*_-based Maxpool exhibits the highest test accuracy after training. To further investigate the efficacy of this algorithm, the time window used for Maxpooling was reduced from 100% of the total number of input image timesteps to 50, 25, and 10%. The results from this experiment are shown in Table 3. Here, we can see that an accurate MaxPool decision can be made with a partial observation window with a marginal reduction in accuracy. Based on this observation, it could be concluded that non-spiking input encoding helps to identify the strongest activations early, since the membrane potential integration starts from the very first timestep, and not just from the very first input spike. Such an approach allows to trade-off fractional temporal data loss for a regeneration of the complete spike sequence for further layers. For example, only the first 10% of timesteps will be lost or require regeneration. As a result, utilizing only the fraction of the temporal data for a MaxPool decision results in higher energy savings, similar to early training termination in SNN, where adequate SNN performance could be achieved using only fraction of output spikes or dataset as a whole (Choi and Park, 2020; Kwak and Kim, 2022). Alternatively, the I&F neuron membrane potential, *V*_*M*_, could be precharged to the winning *V*_*M*_ potential after pooling, thus minimizing data loss. Another minor observation is that training with a smaller pooling window could lead to higher accuracy, as it can be observed in cases of 50 and 25 timesteps window for MNIST. It could be attributed to the stochastic nature of the training algorithm used (AdaDelta), where the network with a smaller pooling window resulted in marginally higher performance by chance.

**Table 3:** Testing accuracy after training on image classification tasks of the Max *V*_*M*_ MaxPool SCNN model with the pooling window less or equal to the number of timesteps.

**MaxPool window, timesteps**	**MNIST (%)**	**Fashion MNIST (%)**	**CIFAR10 (%)**
**Autograd trained with static input**			
100	98.50	91.24	79.49
50	98.10	90.61	78.96
25	98.14	90.25	78.82
10	98.55	89.15	77.75
**Autograd trained with spiking input**			
100	98.26	88.16	74.19
50	97.75	86.83	67.79
25	97.89	86.85	65.64
10	95.70	81.82	58.44

### 5.3. Training with limited weight resolution for the *V*_*M*_ based MaxPool

Neuromorphic NVM devices, such as ReRAMs (or memristors), became popular for actual or theoretical VMM realization on neuromorphic mixed circuit chips. Ideally, NVM devices have infinite conductance resolution and state stability. However, they demonstrate a small number of stable conductance states (Esmanhotto et al., 2020). As a result, we investigated how limited weight resolution would affect classification accuracy after training the Max *V*_*M*_ MaxPool SCNN. Moreover, we trained two versions of the SCNN, one with a 100% MaxPool window and the another with just 10%, so we could observe if the smaller MaxPool window will have negligible classification accuracy degradation in the context of severe device limitations. Limited weight resolution was implemented with *aihwkit* AnalogConv2D and AnalogLinear (Dense) layers with the non-ideal device model. We used the *ConstantStepDevice* behavioral model and bounded the weights to the [−0.5, 0.5] range with the update noise parameter *dw*_*min*_*std* = 0.3 (IBM, 2022b). Furthermore, we defined the minimum weight update δ*w*_*min*_ (the smallest weight increment) dependency on weight resolution in bits, *n*_*bits*_, as


(10) $$\begin{array}{l} \delta w_{min}=\frac{1}{2^{n_{bits}+1}} \end{array}$$

To train the SCNN with analog layers, we modified the PyTorch AdaDelta optimizer to support *aihwkit* analog workflow based on the *analog tiles* (IBM, 2022a). Table 4 shows the classification accuracy results after autograd training with limited weight resolution for 100 and 10% *V*_*M*_ time window. As shown, lowering weights resolution hardly affects MNIST classification accuracy. Even in the most severe case of 1-bit weight and 10% MaxPool window, performance degradation is < 1%. FashionMNIST classification drops significantly at the 4-bits resolution or lower, but overall accuracy stays above 80% even for the 1-bit case. As expected, CIFAR10 is more affected by device limitations. In order to maintain the CIFAR10 accuracy degradation below 10%, at least 6-bit weight resolution is desired for training. However, 2-bit weights are enough for at least 60% accuracy of SCNNs. Comparing MaxPool window sizes, the accuracy difference does not exceed 2.64%, even for the worst case of the 1-bit resolution in CIFAR10.

**Table 4:** Accuracy of the autograd trained SCNN with Max *V*_*M*_ based MaxPool, 100 and 10% time windows and limited weight resolution.

**Weight resolution**	**MNIST (%)**	**Fashion MNIST (%)**	**CIFAR10 (%)**
**100% MaxPooling window**			
Ideal weights	98.50	91.24	79.49
10-bits	98.92	90.14	76.84
8-bits	98.97	89.32	76.95
6-bits	98.63	89.72	73.60
4-bits	98.65	87.71	71.32
2-bits	98.08	85.17	62.78
1-bit	97.60	81.83	48.08
**10% MaxPooling window**			
Ideal weights	98.55	89.15	77.75
10-bits	98.88	89.87	75.88
8-bits	98.97	89.21	74.60
6-bits	98.51	89.59	69.94
4-bits	98.57	87.73	69.64
2-bits	99.33	83.56	61.74
1-bit	97.68	80.83	45.44

## 6. Mixed-signal circuit realization of SCNN

**Figure 7:**
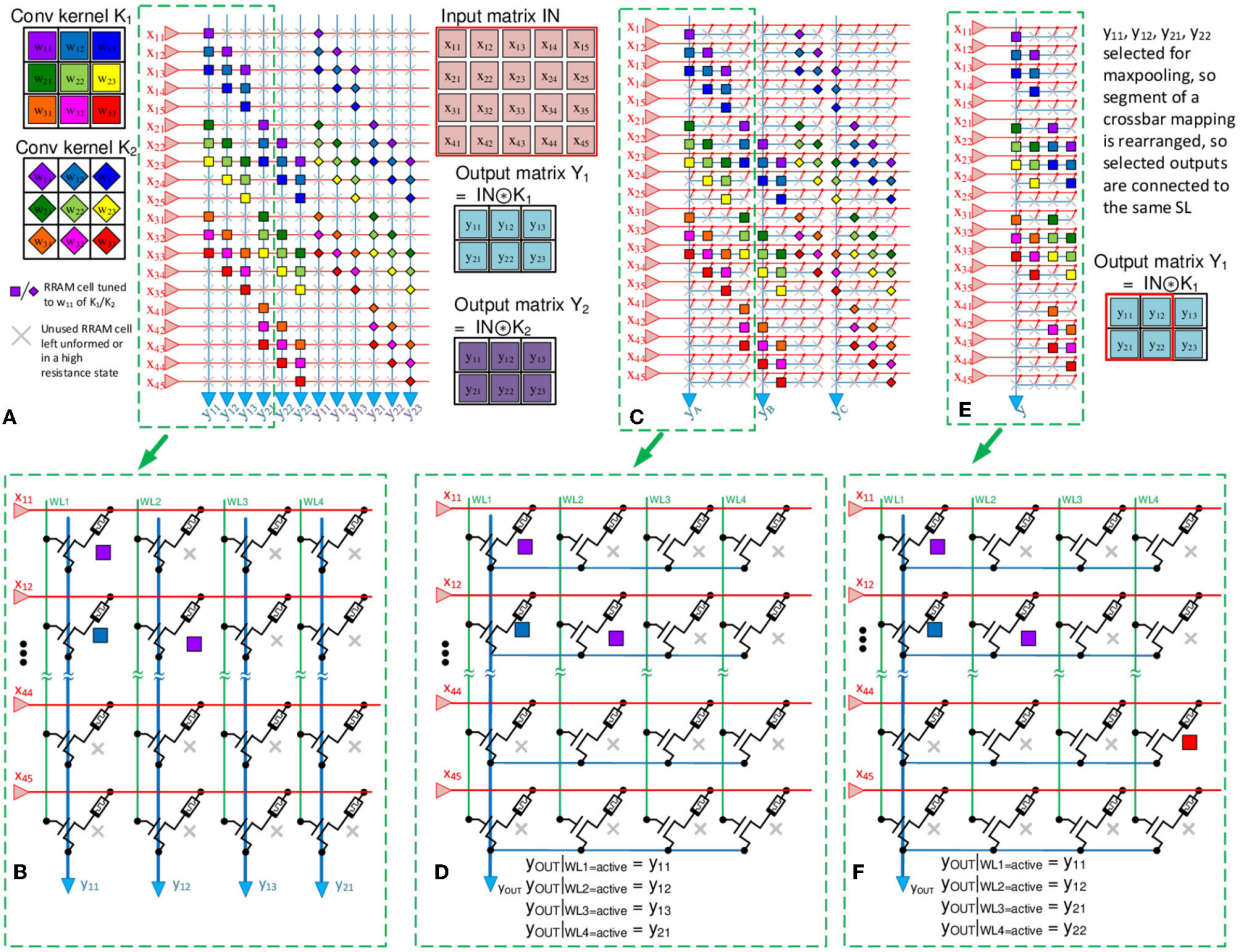
**(A)** Example of Toeplitz mapping of *F* = 2 convolution kernels (3 × 3) on a crossbar for a Conv2D operation on an input of size *M*_*x*_×*M*_*y*_= 5 × 4 (the same weight label, e.g., *w*_1, 1_, could be reused by different convolutional kernels, but the actual weights could be different). **(B)** 1T1R architecture for the selected crossbar segment in **(A)**. The 2T2R architecture will be similar but 1T1R shown for visual clarity. **(C)** Proposed crossbar mapping scheme with temporal Maxpooling with merged SLs/columns. Here, four columns are multiplexed into one readout circuit. **(D)** 1T1R architecture for the crossbar segment, selected in **(C)**. **(E)** Segment of the rearranged multiplexed crossbar scheme, where outputs belonging to the same MaxPool spatial window appear on the same SL/column. **(F)** 1T1R architecture for the crossbar segment, shown in **(E)**.

We now focus on realizing SCNNs and MaxPooling in mixed-signal neuromorphic hardware. Mapping convolutions to an NVM crossbar array is a problem of current interest in CNN-based neuromorphic chips. In recent works, fully-parallel convolutions are realized by “unrolling” the overlap and save operation and mapping it to NVM (ReRAM) arrays much larger than the kernel size, *K*^2^×1 (Gopalakrishnan et al., 2020; Saxena, 2021a). This is accomplished using Toeplitz matrix mapping, as illustrated in Figures 7A, B (Yakopcic et al., 2016; Gopalakrishnan et al., 2020).

Here, the *M*_*x*_×*M*_*y*_×*C* input tensor is flattened, and for *C* = 1, the convolution operation is mapped to a *M*_*x*_*M*_*y*_×*N*_*x*_*N*_*y*_*F* crossbar array with *M*_*x*_*M*_*y*_ inputs and *N*_*x*_*N*_*y*_*F* readout outputs. The inputs are pre-neurons (or DACs in non-spiking VMMs), and the output readout circuits are post-neurons (or ADCs in non-spiking VMMs). Specific readout circuits are discussed later in Section 6.2.

However, Toeplitz mapping underutilizes the NVM array, as several devices are unused with a utilization ratio of


(11) $$\begin{array}{l} \eta =\frac{K^{2}N_{x}N_{y}}{M_{x}M_{y}N_{x}N_{y}}=\frac{K^{2}}{M_{x}M_{y}} \end{array}$$

which is $\frac{9}{20}$ or 45% for the example in Figure 7. An advantage of this scheme is that all convolution outputs are simultaneously available without changing the inputs.

As discussed in Section 4, MaxPooling is challenging to realize at the circuit level without a significant area overhead, compared to subsampling alternatives, such as average pooling (Lin et al., 2014; Iandola et al., 2016) or non-overlapping convolutional kernels (Springenberg et al., 2015; Gopalakrishnan et al., 2020). Moreover, in the case of a fully parallel readout, where each array output is available simultaneously, each column requires its own readout periphery circuit. Due to the nature of a CNN, only one output from a pooling window propagated further. Using Toeplitz CNN array mapping, output rearrangement, and time domain Peak-Detector periphery circuits, fully parallel output access is maintained with a reduced circuit area overhead due to the time-multiplexed periphery circuit utilization.

### 6.1. Area-efficient CNNs using RRAM crossbar array

In the scheme depicted in Figures 7A, B, each select-line (SL) will require an individual readout circuit, i.e., a post-neuron in SCNN or an ADC in regular CNN implementations. Also, the pitch of a crossbar array will depend on the layout size of the post-neurons (or column ADCs), which must be designed in a narrow column area (Khaddam-Aljameh et al., 2021). Alternatively, several NVM (IT1R or 2T2R) columns can be fitted in the pitch of a column readout circuit. For example, a 100 μm readout circuit pitch can accommodate 32 columns at a cell pitch of 3.125 μm. It suggests the possibility of sharing peripheral circuits for array operations, such as convolution readout and pooling, leading to denser area-efficient arrays.

There are several use cases for arrays with peripheral circuit reuse. For example, weights of multiple neural layers can be mapped into a single physical array with appropriate sub-array scheduling (Qiu et al., 2018). Furthermore, in such a configuration, layers outputs or activations could be fed back into the same array, emulating sequential layer-to-layer data flow. Similarly, multiple digital operations could be mapped on a single physical array (James et al., 2020).

We previously proposed the optimized Toeplitz mapping scheme with MaxPooled circuit reuse (Dorzhigulov et al., 2022). This expanded work details the complete mixed-signal SCNN with optimized Maxpooling and circuit blocks reuse. Here, we propose reusing a comparator from the I&F neuron circuit to minimize the effect of temporal data loss due to the time delay required for the MaxPool decision and also to keep the winning neuron active after the pooling decision is made. This approach allows us to implement the proposed membrane potential-based (*V*_*M*_) Maxpooling in CMOS/ReRAM mixed-signal hardware. This scheme is illustrated in Figures 7C, D. Here, *L* = *s*^2^ SLs are multiplexed to a single shared readout circuit in the column. Since outputs within a group are accessed sequentially in the proposed configuration, it essentially allows performing spiking MaxPool in the time domain. With the typical *s*×*s* = 2 × 2 MaxPool window, *s*^2^ = 4 outputs on the SLs, that correspond to the Maxpool window, are wire-ORed together. Each column is accessed sequentially by selecting the corresponding WL, and their outputs are compared for Maxpooling. Thus, each output within a group is read in four WL-select cycles.

Figures 7E, F shows the rearranged array configuration, where outputs belonging to the same pooling window are spatially grouped by connecting them to the respective SL. In addition to readout circuit reuse, with only $\frac{N_{x}N_{y}}{s^{2}}$ readout circuits and higher array area- and energy- efficiency, the MaxPool outputs are available simultaneously for each pooling group, so the MaxPool layer outputs are available for the next layer at the same time. A major drawback of this approach is the temporal data loss due to the output time-multiplexing. However, the proposed Max *V*_*M*_-based MaxPooling is capable of adequate classification accuracy with only a fraction of temporal data. Thus, the effect of this overhead could be minimal for a small enough MaxPool decision time window.

### 6.2. Mixed-signal circuit for readout and temporal MaxPooling

**Figure 8:**
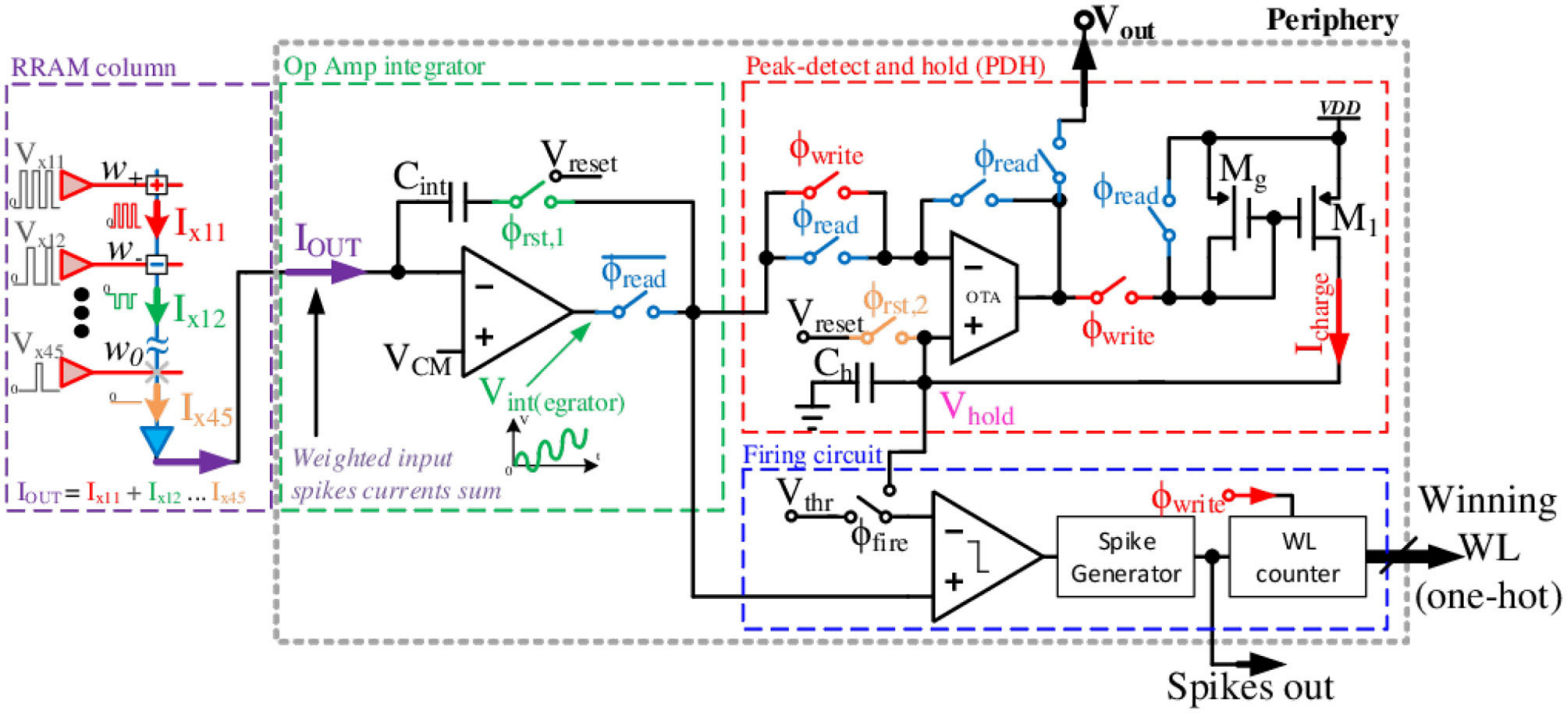
Proposed array readout circuit with integrator and comparator sharing in time-domain followed by the PDH circuit.

We now present the transistor-level circuit details for the shared readout and temporal MaxPooling circuit shown in Figure 8. A crossbar array and peripheral circuits were realized in ST 130 nm H9A CMOS with MAD200 NVM technology with post-fabricated HfO_*x*_ ReRAM in the back-end-of-the-line (BEOL) process. A 2T2R ReRAM crossbar array, similar to the one seen in Figures 3, 7, is used for realizing signed weights. We used the 1.8 V supply analog transistors available in the process. The circuit operation comprises four phases: Integrate, Write, Read, and Resets, controlled by the strobe signals: *WL*_1 − 4_, ϕ_write_, ϕ_reset_, ϕ_rst, 2_, and ϕ_read_. Using the 2 × 2 MaxPool window example from Figure 7, the circuit uses shared integrator op-amp and Peak-Detector-and-Hold (PDH) circuits to identify the highest membrane potential, *V*_*int*_≜*V*_*M*_, from a group of four-column outputs.

**Figure 9:**
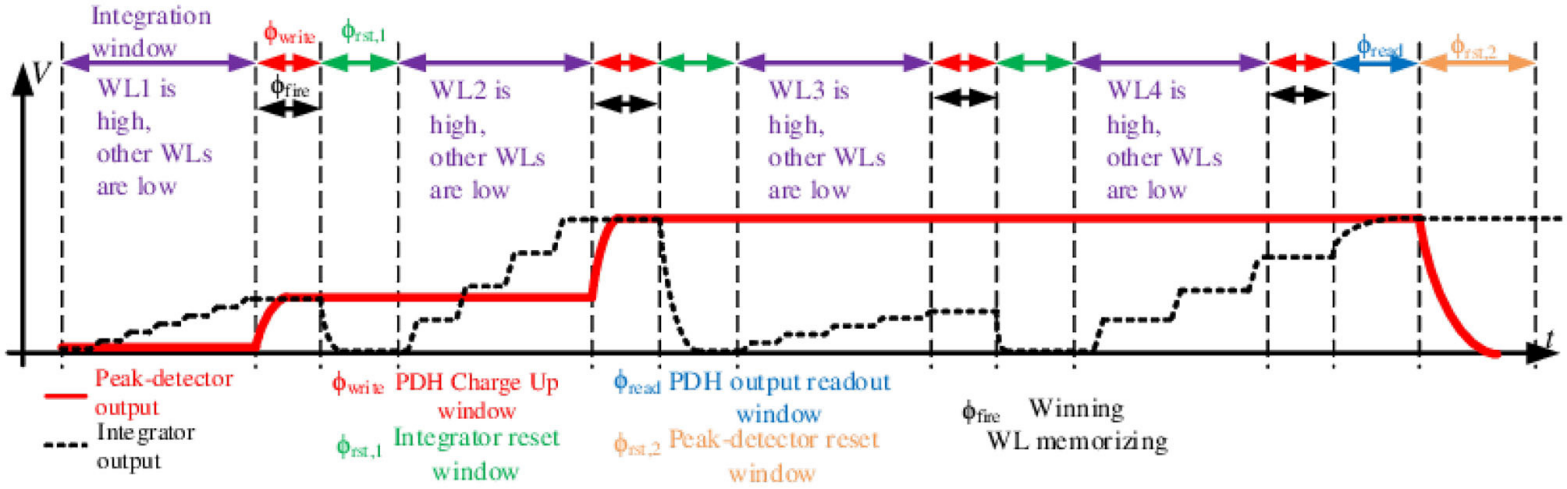
Timing diagram of the proposed periphery MaxPool circuit. 2 × 2 MaxPool window (thus four WLs) and no negative weights (integrator output only increasing) assumed for illustration.

The circuit timing diagram is shown in Figure 9. The sequence starts with the integrator reset and selecting WL_1_ to be high. The op-amp integrator integrates the incoming weighted spikes from the *K*^2^ active inputs in the selected column over time. This recovers the analog information of the VMM output through low-pass filtering (integration), which is analogous to the membrane potential, *V*_*M*_, in an I&F neuron. Next, as ϕ_write_ goes high, the PDH circuit samples and holds the integrator output as *V*_*hold*_ on the capacitor *C*_*h*_. Following the writing phase, the integrator resets with the ϕ_reset_ strobe.

Then the same cycle repeats, but with WL_2_ going high and so on. The only difference is that the PDH circuit compares the new integrated sample with the previously held voltage value and only retains the highest voltage. Here, *V*_*int*_ is on the negative input of the operational transconductance amplifier (OTA), where *V*_*int*_>*V*_*hold*_ charges up capacitor *C*_*h*_ through the current mirror (De Geronimo et al., 2002). If *V*_*int*_ drops below *V*_*hold*_ on *C*_*h*_, *V*_*hold*_ remains unchanged until *V*_*int*_>*V*_*hold*_ again.

At the end of all WL cycles, the ϕ_read_ strobe is asserted. The read phase sets the OTA in a voltage follower configuration, making *V*_*out*_ = *V*_*hold*_. In that phase, the *C*_*int*_ capacitor is also charged to a *C*_*h*_ potential, making *C*_*int*_ carry the pooled potential to the next I&F stage. Such an approach allows the preservation of the spiking data from the MaxPool decision phase in the form of the saved integrated potential of the winning input. The PDH reset strobe, ϕ_rst, 2_, resets *V*_*hold*_ to *V*_*reset*_, so *V*_*int*_ below *V*_*reset*_ will not be detected. Thus, it effectively acts as an implicit ReLU. Thus, *V*_*reset*_ serves as the ReLU bias. The maximum pooled value is read out as *V*_*out*_. This voltage can be digitized using an ADC for non-spiking VMM or converted to spikes using an I&F neuron circuit.

In addition, it is feasible to reuse a comparator, an essential part of the I&F CMOS neuron (Wu et al., 2015b). The primary function of the comparator is to signal when the integrated potential *V*_*M*_ exceeds the firing threshold *V*_*thr*_. With additional strobe ϕ_fire_, the comparator input can be switched to trigger a signal when *V*_*int*_ is greater than *V*_*hold*_. That trigger signal activates a digital memory to store a currently active WL as a MaxPool winner. The digital counter, clocked by ϕ_write_, tracks the currently active WL. Then, the winning WL is encoded in a digital N-bit one-hot output, where N is the total number of WLs. One-hot encoding keeps only the winning WL high (“1”), thus passing spikes only from the MaxPool winner. At the end of each time-frame, the circuit resets to select and propagate a new winner for the next frame. Finally, by implementing the MaxPool operation with partial temporal information, as in Section 5.2, the WL_*j*_ strobe width can be reduced.

Combining the optimized Toeplitz mapping scheme with shared peripheral circuits with implicit ReLU, we can implement fully-parallel convolutions, MaxPooling, and nonlinearity in an area-efficient manner. Thanks to the synchronous nature of the proposed design (i.e., cycling through the WLs in a predefined MaxPool time window), we can implement the explicit Max *V*_*M*_-based MaxPool scheme, proposed and evaluated in software earlier in Section 5.

### 6.3. Circuit simulation results

**Figure 10:**
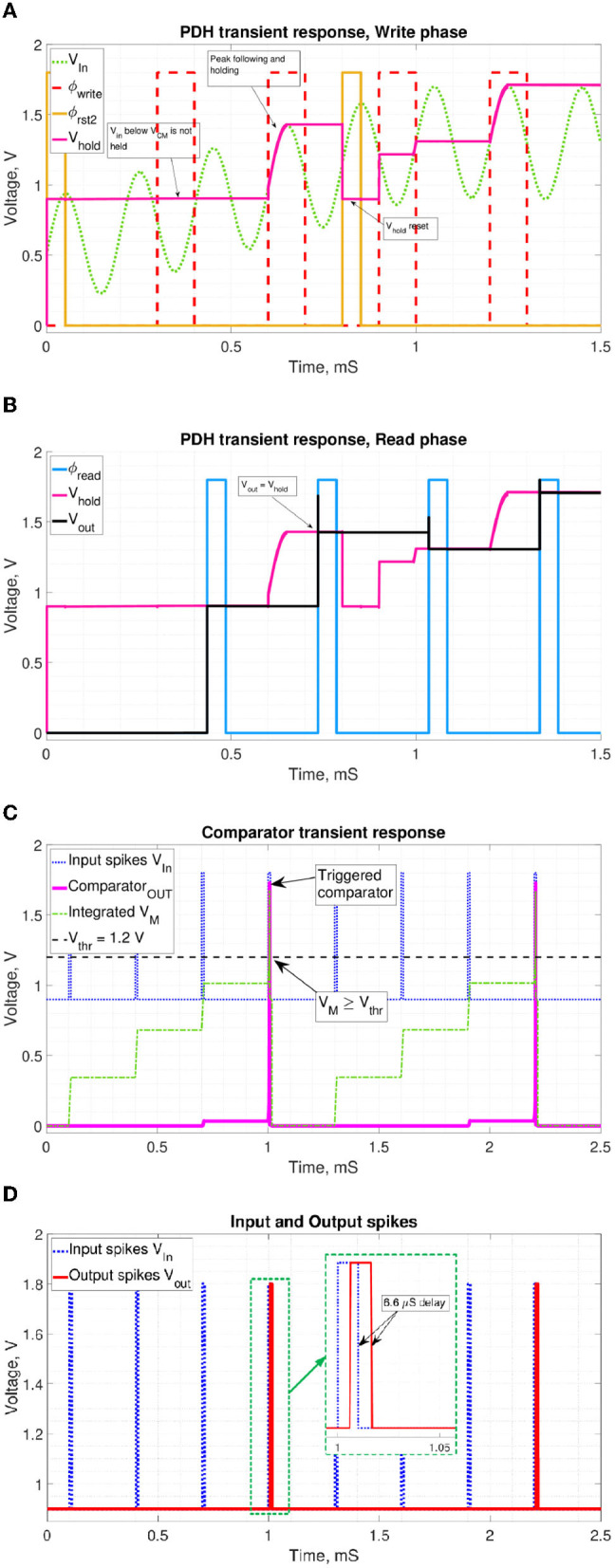
Transient simulations of the proposed periphery. **(A)** PDH write and reset phases. ϕ_*write*_ enables charging up of *C*_*h*_, while ϕ_*rst*, 2_ resets it to *V*_*rst*_ = 0.9*V*. **(B)** PDH read phase. ϕ_*read*_ enables the OTA to set *V*_*out*_ = *V*_*hold*_. **(C)** Comparator phase. *V*_*M*_ reaching *V*_*thr*_ triggers the comparator circuit. **(D)** Input and output spikes. Observed delay depends on the *V*_*M*_ state before firing.

The transistor-level circuits were simulated using Cadence Specter and 130 nm CMOS/OxRAM device models for *V*_*DD*_ = 1.8*V*. Figure 10A shows a transient simulation of the PDH circuit functionality during the write and reset phase. When the signal ϕ_*write*_ is high, *C*_*h*_ charges up to the *V*_*int*_ level, and if *V*_*hold*_ across the *C*_*h*_ capacitor is below *V*_*int*_, it works as the PDH circuit. ϕ_*rst*2_ resets *C*_*h*_ to the *V*_*reset*_ level, set to *V*_*CM*_ = 0.9*V* of the OTA. Thus, *C*_*h*_ will not charge below the *V*_*CM*_ level, effectively functioning as a ReLU.

Figure 10B shows that when ϕ_*read*_ is high, *V*_*out*_ is pulled to the *V*_*hold*_ level. Figure 10C demonstrates the comparator response to increasing *V*_*M*_ as the input spikes are integrated over time. *V*_*M*_ crossing of a threshold voltage triggers the comparator response. Figure 10D shows the fired output spike following the triggering input spike with a slight dynamic delay.

Circuit simulation shows the total bias current of *I*_*bias, DC*_ = 1.06μ*A* or 1.9 μW power consumption. The SCNN circuit realization incurs 0.85 pJ energy per synaptic operation (synOp). We estimated the average number of spikes used for performing an inference based on Equation (12) described in Rueckauer et al. (2017), where *t* is the current time step, *T* is the total simulation duration, *l* is the current layer, *L* is the total number of layers, *s*_*l*_ is the number of fired spikes in the layer *l* at the time *t*. *f*_*out*_ denotes fan-out, the total number of outgoing synaptic connections to the subsequent layer. The SCNN results in 21.7 μJ per inference for the CIFAR10 dataset. However, considering the static energy dissipation from the peripheral circuits and additional delay caused by MaxPool layers (10% per layer), this metric increases to 181 μJ per inference.


(12) $$\begin{array}{l} \overset{T}{\underset{t=1}{\sum }}\left[\overset{L}{\underset{l=1}{\sum }}f_{out,l}s_{l}\left(t\right)\right]=\text{synOps/image} \end{array}$$

A common benchmarking metric for AI hardware is the throughput or the number of operations per second per Watt (OPS/W), which is computed as OPS = MACs per inference × Frequency, where each multiply-and-accumulate (MAC) accounts for two operations (Sze et al., 2020). MACs per inference can be calculated according to Equation (13) (Rueckauer et al., 2017), where fan-in *f*_*in, l*_ is defined as the number of incoming connections to a neuron, and *n*_*l*_ is defined as the number of neurons in the *l* layer.


(13) $$\begin{array}{l} \overset{L}{\underset{l=1}{\sum }}\left(2f_{in,l}+1\right)n_{l}=\text{MACs/image} \end{array}$$

For a 50 μ*s* timestep and pipelined sequencing of SCNN arrays for each SNN layer with 100 timesteps per inference, the estimated throughput for the SCNN is 34.4 GOPS for the MNSIT/FashionMNIST network and 85.6 GOPS for the CIFAR10 network. This corresponds to an equivalent energy-efficiency metric of 234.4, 236.5, and 473.6 TOPS/W for MNIST, FashionMNIST, and CIFAR10 networks, as shown in Table 5. It also includes the energy-efficiency metrics comparison with some of the recent compute-in-memory chips. As can be seen, we can obtain competitive results for pJ/SOp compared to SNN chips and TOPS/W compared to ReRAM-based image classification chips.

**Table 5:** Energy consumption estimation and benchmarking for the SCNN with the Max *V*_*M*_ potential based MaxPool with a 10% window.

**Dataset**	**I&F layer 1 average count spike fires**	**I&F layer 2 average count spike fires**	**I&F layer 3 average count spike fires**	**MOps/image**	**pJ/synOp**	**μJ per inference**	**TOPS/W**
**This work**							
MNIST	47,621	11,910	1,965	27.52	0.85	147^†^	234.4
FashionMNIST	46,682	9,595	2,306	25.72	0.85	146^†^	236.5
CIFAR10	41,849	12,898	4,098	25.55	0.85	181^†^	473.6
**SNN implementations**							
Valentian et al. (2019)							
ReRAM-based one-layer SNN for MNIST classification					17–180	–	–
Frenkel and Indiveri (2022)							
Spiking RNN in 28 nm FDSOI SRAM for gesture recognition					5.3	46.1	–
Wang et al. (2021)							
SNN in 65 nm for keywords spotting					1.5	–	–
Liu et al. (2022)							
Asyncronous SNN in 180 nm for ECG classification					0.53	–	–
**ReRAM compute-in-memory macros for data classification**							
Liu et al. (2020)							
Analog ReRAM-based two-layer perceptron for MNIST classification					–	–	78.4
Chang et al. (2022)							
General purpose binary ReRAM/SRAM-based compute-in-memory 40 nm macro					–	–	26.56
Hung et al. (2022)							
ReRAM-based compute-in-memory macro in 22 nm for CIFAR10 classification					–	–	61.8

^†^Energy consumption estimates based on the crossbar array and neural peripheral circuits.

## 7. Discussion and future work

In this work, we have proposed three variations of MaxPool algorithms for SCNNs. The benefits of the proposed algorithms are: (i) temporal MaxPool allows reuse of peripheral circuits, thus minimizing chip area and power, (ii) decision-making based on the partial temporal information is possible, implying that the entire duration of the spike-encoded image is not required to perform MaxPooling a correct output.

While mixed-signal SCNN circuit design was performed and block-level circuits simulated at the transistor level, a higher software abstraction is necessary for evaluating system-level performance for the entire SCNN. We developed a customized software pipeline to simulate spiking CNNs using 4D tensors. The aihwkit framework allowed us to simulate the effect of the device-level non-idealities on SCNNs classification accuracy. Next, we evaluated the proposed MaxPool algorithms by training spiking variations of the baseline CNN using PyTorch and its native auto-differentiation functionality.

As can be seen from the results for the CIFAR10 dataset, the difference between SCNN converted from ideal CNN and proposed *V*_*M*_-based MaxPool SCNN is 1.07%. Since such a result was obtained using training, as opposed to transfer learning, it highlights the prospects of proposed algorithms for on-chip training.

To further support the advantages of the proposed *V*_*M*_ MaxPool in a mixed-signal neuromorphic chip, we implemented the desired functionality with a novel shared readout circuit, effectively implementing Convolutional Layer, MaxPool, and ReLU in a single area and energy-efficient circuit block. The proposed periphery circuit could be even more energy efficient if the same op-amp and OTA could be reused for multiple operation phases (Wu et al., 2015a). There is further scope to increase array utilization for CNNs by employing more sophisticated row and/or column multiplexing in time. Lastly, energy-efficiency estimation of the proposed array and periphery shows competitive results compared to contemporary CiM chip designs.

Moreover, our work shows the possibility of utilizing native backprop-based training algorithms for SCNNs. Even though the topic of on-chip learning is highly convoluted, some researchers are experimenting with mixed-signal circuits to train ANNs directly on a neuromorphic chip with Backprop (Greenberg-Toledo et al., 2019; Krestinskaya et al., 2019). Based on our results, we can also consider using similar training circuits for Spiking ANNs.

The future continuation of this research work can entail the investigation of on-chip learning and optimizing the impact of spike encoding error on the overall classification performance of the network.

## Data availability statement

The raw data supporting the conclusions of this article will be made available by the authors, without undue reservation.

## Author contributions

AD developed the experimental procedures, implemented and simulated the methods, and drafted the manuscript. VS contributed to the methodology development and manuscript edits. All authors contributed to the article and approved the submitted version.
